# Focus on advanced inorganic materials science: non-traditional concepts and approaches

**DOI:** 10.1088/1468-6996/16/2/020302

**Published:** 2015-04-01

**Authors:** Mitsuru Itoh, Takashi Akatsu, Masaki Azuma, Keigo Kamata, Toshio Kamiya

**Affiliations:** Tokyo Institute of Technology, Japan

Advanced inorganic materials have been used in the worldwide economic development over the past century and stimulated current research activities in many emerging research topics ranging from fundamental science to applications in sustainable technology, energy conversion, and environmental issues. Advanced ceramics, including optical, electronic, catalytic, biological, medical, and environmental materials, contribute substantially to the quality of our lives. However, many challenges remain to be solved in resource, energy, and environmental problems, which require multidisciplinary integration beyond the boundaries of physics, chemistry, engineering, and materials science. In this context, creation of novel advanced materials with innovative functions under the concept of ‘element strategy’ has received significant attention in recent years. For example, this concept has been used to develop alternatives to materials that contain rare metals, such as Nd and Dy based magnets in high-power motors of hybrid electric vehicles. Furthermore, computational methods will play an important role in predicting and understanding the structure–property relationship and in the design of novel functional materials. In this focus issue, we call these strategies ‘non-traditional concepts and approaches’.

This focus issue aims to review the current trends in ‘advanced inorganic materials science: non-traditional concepts and approaches’. It is related to the annual *International Conference on the Science and Technology for Advanced Ceramics* (STAC). Within the concept of ‘element strategy’, Li and Sakka review Gd_3_Al_5_O_12_-related materials and describe phosphors and transparent ceramics that may replace indium-containing compounds, such as InSnO_2_. In an example of a theoretical approach, Chang and co-authors applied first-principles molecular dynamics simulations of oxygen vacancy diffusion to amorphous In–Ga–Zn–O thin-film transistors, aiming to avoid hole traps that degrade the device characteristics. Omata and co-authors reviewed the guiding principles of wurtzite-derived ternary I–III–O_2_ semiconductors (for instance, LiGaO_2_) and their band-engineering, and Yamaura and co-authors applied high pressures to KSbO_3_-type Os compounds and compared their magnetic and electronic properties with theoretical predictions.

Environmental issues are also an important topic in advanced inorganic materials science. Catalytic reduction of NO with NH_3_ over a TiO_2_ photocatalyst was studied by Teramura *et al*. Nakagawa and co-authors reported a highly active and selective supported bimetallic rhodium–molybdenum catalyst for the hydrogenation of amides, especially with the addition of CeO_2_. Hara and co-authors presented a comprehensive review on the recent progress in solid catalysts for the conversion of biomass into valuable chemicals. One of the highlights of this focus issue is a review of negative thermal expansion induced by intermetallic charge transfer at room temperature presented by Azuma *et al.* Another highlight is a review article on reinforcing ceramics with carbon nanotubes for structural applications presented by Estili and Sakka.

We hope that this focus issue will contribute to the understanding of advanced ceramics and bridge the gap between fundamental research and technological developments.

